# Effects of exercise in breast cancer patients: implications of the trials within cohorts (TwiCs) design in the UMBRELLA Fit trial

**DOI:** 10.1007/s10549-021-06363-9

**Published:** 2021-08-24

**Authors:** Roxanne Gal, Evelyn M. Monninkhof, Carla H. van Gils, Rolf H. H. Groenwold, Sjoerd G. Elias, Desirée H. J. G. van den Bongard, Petra H. M. Peeters, Helena M. Verkooijen, Anne M. May

**Affiliations:** 1 Julius Center for Health Sciences and Primary Care, University Medical Center Utrecht, Utrecht University, STR 6.131, PO Box 85500, 3508 GA Utrecht, the Netherlands; 2 Department of Clinical Epidemiology, Leiden University Medical Center, Leiden, The Netherlands; 3 Department of Radiation Oncology, Amsterdam University Medical Centers, Amsterdam, The Netherlands; 4 Division of Imaging and Oncology, University Medical Center Utrecht, Utrecht University, Utrecht, The Netherlands

**Keywords:** Trials within cohorts, TwiCs, Breast cancer, Physical activity, Quality of life, Fatigue

## Abstract

**Purpose:**

The Trials within Cohorts (TwiCs) design aims to overcome problems faced in conventional RCTs. We evaluated the TwiCs design when estimating the effect of exercise on quality of life (QoL) and fatigue in inactive breast cancer survivors.

**Methods:**

UMBRELLA Fit was conducted within the prospective UMBRELLA breast cancer cohort. Patients provided consent for future randomization at cohort entry. We randomized inactive patients 12–18 months after cohort enrollment. The intervention group (*n* = 130) was offered a 12-week supervised exercise intervention. The control group (*n* = 130) was not informed and received usual care. Six-month exercise effects on QoL and fatigue as measured in the cohort were analyzed with intention-to-treat (ITT), instrumental variable (IV), and propensity scores (PS) analyses.

**Results:**

Fifty-two percent (*n* = 68) of inactive patients accepted the intervention. Physical activity increased in patients in the intervention group, but not in the control group. We found no benefit of exercise for dimensions of QoL (ITT difference global QoL: 0.8, 95% CI = − 2.2; 3.8) and fatigue, except for a small beneficial effect on physical fatigue (ITT difference: − 1.1, 95% CI = − 1.8; − 0.3; IV: − 1.9, 95% CI = − 3.3; − 0.5, PS: − 1.2, 95% CI = − 2.3; − 0.2).

**Conclusion:**

TwiCs gave insight into exercise intervention acceptance: about half of inactive breast cancer survivors accepted the offer and increased physical activity levels. The offer resulted in no improvement on QoL, and a small beneficial effect on physical fatigue.

**Trial registration:**

Netherlands Trial Register (NTR5482/NL.52062.041.15), date of registration: December 07, 2015.

## Introduction

Fatigue is the most often reported side-effect of breast cancer and its treatment, which can persist for years and negatively affect quality of life (QoL) [1–3]. Meta-analyses of randomized controlled trials (RCTs) have shown that physical exercise has positive effects on fatigue and QoL in patients with cancer [4–8]. However, these effects were often small, which may be partly due to the inability of blinding the intervention [9]. Patients who decided to participate are generally motivated to exercise and may be disappointed when allocated to the control group. Consequently, they drop-out or start exercising by themselves, the latter resulting in contamination and dilution of the intervention effect [10]. Other disadvantages of conventional RCTs are time-consuming accrual and inclusion of a selective study sample [11, 12].

The Trials within Cohorts (TwiCs) design, also known as the cohort multiple randomized controlled trial (cmRCT) design, was proposed as an alternative to conventional pragmatic RCTs, and has the potential to overcome above mentioned challenges [13–15]. Using this design, the intervention study is performed within a prospective cohort. Compared to conventional RCTs, the TwiCs design can lead to more efficient patient recruitment, generalizability of results may be improved, and TwiCs allows evaluation of patients’ acceptability of the intervention [14–17].

The current UMBRELLA Fit study is the first trial applying the TwiCs design in the field of exercise-oncology. UMBRELLA Fit examined the effect of a 12-week supervised exercise intervention on QoL and fatigue. Moreover, we aimed to evaluate the applicability of the TwiCs design in the field of exercise-oncology and the implications of the TwiCs design on effect estimation and interpretation of the results.

## Materials and methods

### Study design and procedures

**Fig. 1:**
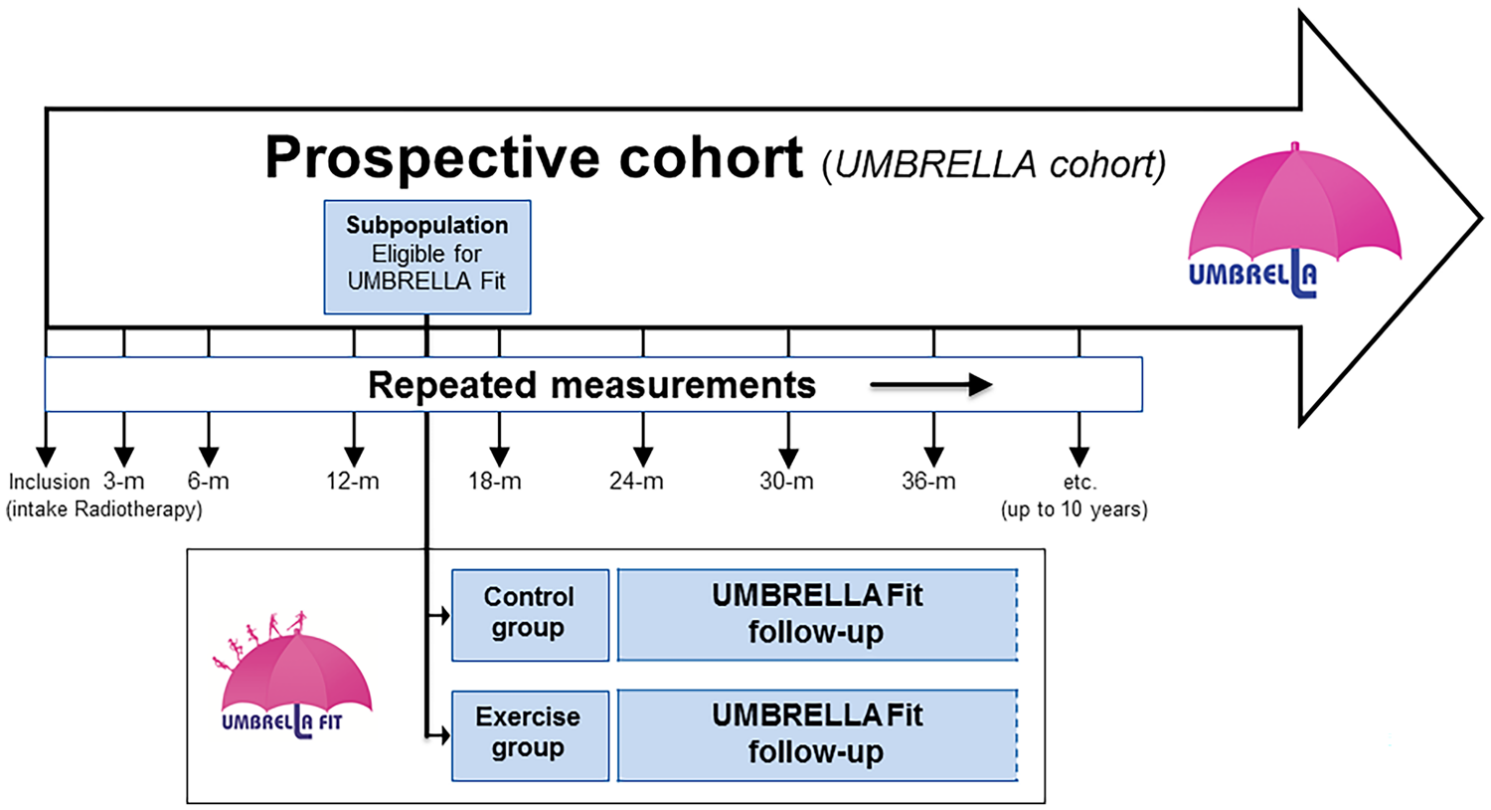
The UMBRELLA Fit trials within cohorts (TwiCs) design ( adapted from Relton et al.)

The study was approved by the Ethics Committee of the University Medical Center Utrecht (UMCU). The UMBRELLA Fit study is a pragmatic, two-arm RCT using the TwiCs design and is conducted within the ‘Utrecht cohort for Multiple BREast cancer intervention studies and Long-term evaLuAtion’ (UMBRELLA) [13, 18, 19]. Since September 2013, all patients with breast cancer who are referred for radiation treatment to the UMCU were invited to participate in the UMBRELLA cohort. At enrollment, first stage informed consent is asked for collection of clinical data and patient reported outcome (Fig. 1) [20]. Additionally, patients could provide broad consent for randomization to future intervention studies. When allocated to an intervention arm (in the future), they will be offered the intervention and asked for (second stage) informed consent when accepting the intervention. Patients allocated to the control arm were not notified about the study and their cohort data were used to estimate intervention effectiveness.

UMBRELLA Fit recruited from October 2015 to March 2018. Women participating in the UMBRELLA cohort meeting the UMBRELLA Fit inclusion criteria (see below) were randomly allocated to either intervention or control with a 1:1 ratio, stratified by time since cohort enrollment (12 or 18 months). Randomization was performed by an independent data manager, using a computer-generated randomization list. Patients allocated to the exercise intervention group received an offer by mail to participate and were contacted by telephone to further explain the study. Patients who refused the offer received usual care. When patients refused because of bad timing of the offer, they got the option to start the exercise program at a later stage. Patients allocated to the control group were not informed about the study, and received usual care. After study completion, all patients in the UMBRELLA cohort, irrespective of participation in this study, were informed by a newsletter.

### Participants

Patients eligible for the UMBRELLA cohort [19], were eligible for UMBRELLA Fit when meeting the following inclusion criteria: (1) broad informed consent for randomization to future intervention studies; (2) 18–75 years of age; (3) completion of the 12- or 18-month cohort questionnaire; (4) cancer treatment completed (except for hormonal treatment); and (5) a physically inactive lifestyle (< 150 min/week performing moderate-to-vigorous leisure time and sports activities).

### Intervention

The 12-week supervised exercise intervention comprised two weekly 1-h combined aerobic and resistance training sessions at a physiotherapist center close to the patient’s home (see details in the supplement). Patients were also encouraged to be physically active with moderate intensity for at least 30 min all days [21].

### Outcome measures

Outcome measures were obtained from routine UMBRELLA cohort measurements. The questionnaire that was completed either 12 or 18 months after cohort enrollment served as baseline and the cohort questionnaire 6 months later was used as follow-up measurement.

#### Quality of life

Quality of life (primary outcome) was measured using the Global QoL score and the five functional scales of the EORTC QLQ-C30 [22]. A QLQ-C30 summary score was calculated using all functional and symptom subscales except global QoL and financial impact [23].

#### Fatigue

Fatigue was measured with the multidimensional fatigue inventory (MFI-20), a 20-item questionnaire containing five dimensions: general fatigue, physical fatigue, mental fatigue, reduced motivation, and reduced activity [24].

#### Anxiety and depression

Anxiety and depression was measured with the validated Dutch version of the hospital anxiety and depression scale (HADS), containing seven items for the depression subscale and seven items for the anxiety subscale [25].

#### Physical activity

Physical activity during an average week in the past months was assessed using the Short QUestionnaire to ASsess Health enhancing physical activity (SQUASH) [26]. A total score was calculated by summing up the minutes per week spent in commuting activities (cycling), leisure time walking and cycling, and sports activities, all with ≥ 4 MET.

#### UMBRELLA Fit measurements

Patients participating in the exercise intervention visited the UMCU pre- and 12-week post-intervention for additional measurements, i.e., questionnaires, cardiopulmonary exercise testing, intervention acceptance and compliance (see details in the supplement) [18].

### Sample size calculation

A required sample size of 166 patients was estimated based on an expected acceptance rate of 70% in the intervention group and a clinically relevant 10-point difference in global QoL (power of 80% and two-sided alpha of 0.05) [18, 27, 28]. After recruitment of 152 patients, the actual acceptance rate was lower than expected (i.e., 55% instead of 70%) and the sample size was updated, as recommended by Candlish et al. to 260 patients [29]. This sample size adaptation was solely based on the acceptance rate. No interim analysis of the trial outcome was performed.

### Statistical analysis

The statistical analyses were specified in our study protocol, which was approved by the Ethics Committee before recruitment started (NL52062.041.15). The statistical analyses plan remained unchanged, but since the TwiCs design is relatively new and little was known about the analyses methods, methods were further refined throughout the analyses process, which was also one of the aims of the current study and which was stated in the statistical analysis plan.

Baseline characteristics and within-group changes are described for all patients and separately for patients who accepted (including patients who withdrew from the intervention in a later phase) and patients who refused the intervention.

#### Within-group changes

Mean changes and corresponding 95%CI in QoL, fatigue, anxiety and depression, and physical activity level from baseline to 6 months follow-up (cohort measurements) and from pre- to 12-week post-intervention (exercise group) were calculated.

#### Between-group differences

ITT linear regression analysis was used to assess between-group differences. ITT analysis might lead to an underestimation of the intervention effect because of intervention refusal [30]. Therefore, we also performed instrumental variable IV analysis using the two-stage least squares method to account for possible non-acceptance in the intervention group as a sensitivity analysis [29, 31]. In the first stage, the relation between treatment assignment and treatment acceptance (compliance) was estimated [32]. In the second stage, the effect of the exercise program on the outcome was estimated, using the predicted values from the first stage as an independent variable in a linear regression model. In the ITT and IV analyses, missing values on covariates and baseline measures of the outcome were multiply imputed (15 imputed datasets using the R mice algorithm [33, 34]), whereas patients with missing outcome values were omitted [35].

In the IV analysis, we could not rule out that intervention refusers were influenced by the offer of the intervention [36]. Therefore, we performed propensity score (PS) analysis as a second sensitivity analysis. Here we estimated the effect in intervention accepters by comparing them to control patients who would have accepted the exercise intervention if offered. To this end, first, propensity scores were estimated for patients in the intervention group using a logistic regression model, i.e., the probability of accepting the intervention, given their observed characteristics (baseline measures of the outcome, age, time since diagnosis, BMI, education). Second, based on the propensity scores, intervention accepters were matched to potential accepters in the control group (i.e., patients who would have accepted the intervention if offered) in a 1:1 ratio without replacement using nearest neighbor matching. In the matched sample, balance of covariates between intervention groups was assessed by means of standardized mean differences and by checking the C-statistic of a refitted propensity score model, indicating the ability of the model to predict treatment status. Finally, linear regression analysis was performed. For PS analysis, missing values of the outcomes were also imputed [33].

All models were adjusted for baseline measures of the outcome, age, time since diagnosis, BMI, and education.

We did not adjust for multiple testing, partly because of multicollinearity between outcomes [37]. Therefore, we reported for all secondary endpoints the effect estimates with 95% confidence intervals and the inferences drawn may not be reproducible.

#### Sensitivity analysis

We repeated the ITT and IV analyses where missing values of the outcome also were imputed. Additionally, ITT analysis was repeated replacing the cohort measurements by the pre- and 12-week post-intervention measurements for patients who did not yet start or did not yet complete the intervention before the cohort follow-up measurement (Fig. 3). Furthermore, PS analysis was performed in refusers (i.e., those who were offered the intervention, yet refused it) to check whether there was an effect of offering the intervention in refusers.

We used SPSS version 25.0 or R Statistical Software version 3.5.1. For all models, model fit was checked and was satisfying.

## Results

### Patients and participation

**Fig. 2:**
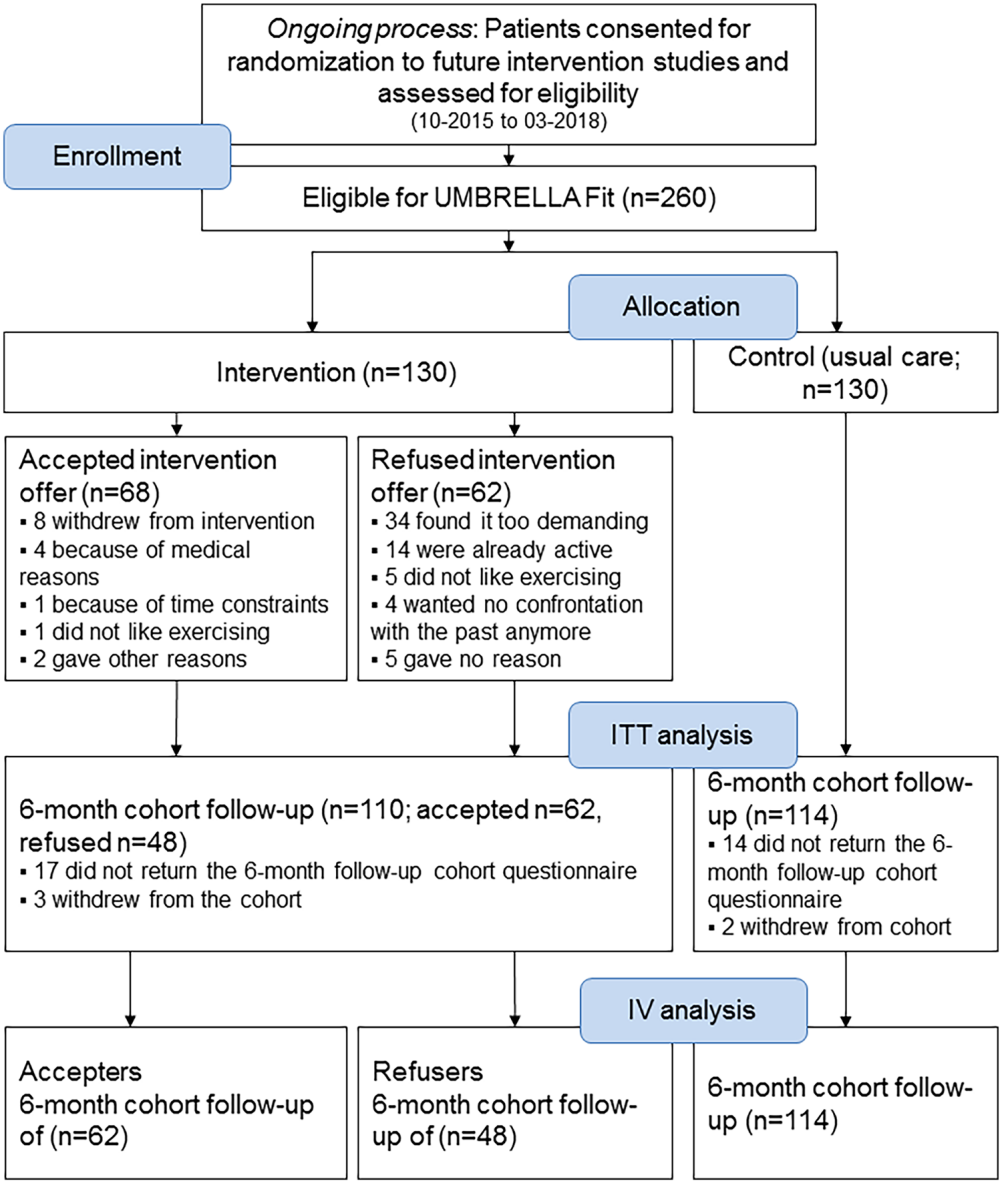
Flow diagram of recruitment, randomization, and follow-up in the UMBRELLA Fit study

In total, 260 patients were randomly allocated to the intervention (*n* = 130) or control group (*n* = 130; Table 1, Fig. 2). Of the patients allocated to the intervention arm, 52% accepted the intervention (*n* = 68). Patients who accepted were slightly younger, had a lower BMI, and were higher educated than refusers (Table 1).

**Table 1:** Baseline characteristics by randomization group and of patients who either accepted or refused the offer of the intervention

	Control (*n* = 130)	Intervention (*n* = 130)	Intervention accepted (*n* = 68)	Intervention refused (*n* = 62)
Age (years), mean (SD)	58.3 (9.5)	58.0 (9.8)	56.6 (9.8)	59.4 (9.6)
Time since cohort inclusion (weeks), mean (SD)	65.9 (13.0)	65.6 (13.1)	66.6 (13.5)	64.5 (12.7)
Body weight (kg), mean (SD)	74.8 (15.2)	74.9 (15.7)	73.6 (13.7)	76.5 (17.8)
BMI, mean (SD)	26.3 (5.0)	26.6 (5.4)	26.0 (4.6)	27.3 (6.2)
Education, *n* (%)				
Primary or (post-)secondary school	71 (56)	80 (64)	39 (59)	41 (71)
College or university	57 (44)	44 (36)	27 (41)	17 (29)
Smoking, *n* (%)				
Never	59 (46)	47 (38)	27 (41)	20 (35)
Ex-smoker	47 (37)	59 (48)	31 (47)	28 (48)
Current smoker	22 (17)	18 (15)	8 (12)	10 (17)
Breast cancer stage, *n* (%)				
In situ/DCIS	16 (14)	20 (17)	12 (19)	8 (14)
Stage I	62 (54)	52 (44)	28 (45)	24 (42)
Stage II	33 (29)	41 (35)	18 (29)	23 (40)
Stage III	4 (4)	6 (5)	4 (7)	2 (4)
Treatment, *n* (%)				
Chemotherapy	42 (37)	50 (42)	27 (44)	23 (40)
Hormonal therapy	46 (40)	48 (40)	23 (37)	25 (44)

*BMI* body mass index, *DCIS* ductal carcinoma in situ, *SD* standard deviation

No differences between patients who accepted and refused the offer of the intervention were observed

Information on body weight, BMI, education, and smoking available for *N*_control_ = 128, *N*_intervention_ = 124, *N*_accepters_ = 66, *N*_refusers_ = 58

Information on breast cancer stage and treatment available for *N*_control_ = 115, *N*_intervention_ = 119, *N*_accepters_ = 62, *N*_refusers_ = 57

#### Adherence

Eight patients withdrew from the intervention after a median number of four training sessions (range 1–15). The 60 patients who completed the exercise intervention attended on average 92% (SD = 9.9) of 24 sessions. At baseline, intervention accepters had a mean VO_2peak_ of 23.8 ml/min/kg (SD = 5.4), which increased, on average, with 1.8 ml/min/kg (95% CI = − 0.4; 0.4) following the exercise intervention.

**Fig. 3:**
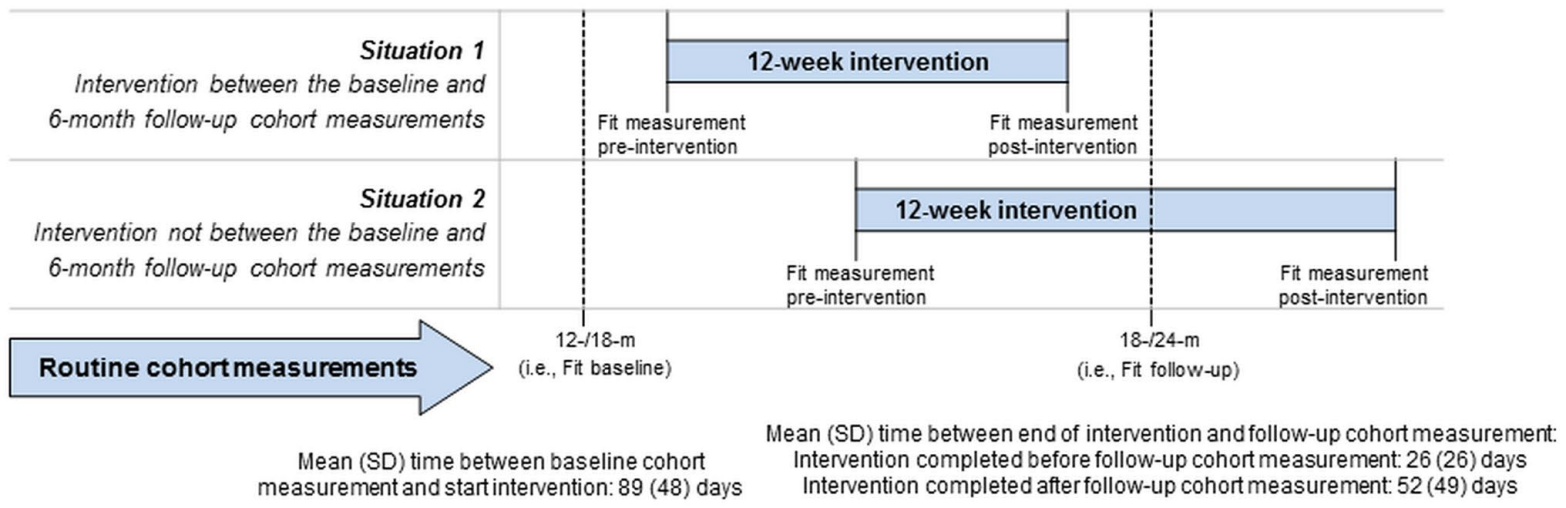
Timing of the intervention (and hence the pre- and 12-week post measurements) relative to the cohort measurements

Timing of the cohort measurements was fixed (Fig. 3). Consequently, about half (n = 35/68) of the patients completed the follow-up cohort questionnaire before intervention completion, whereof three patients started the intervention after the follow-up cohort questionnaire. Reasons for a delayed intervention start were planned vacations, physical conditions (e.g., elective heart surgery), family issues, and time constraints. Eleven intervention (16%) accepters did not complete the follow-up questionnaire.

#### Retention

The 6-month follow-up cohort questionnaire was returned by 87% of the patients. Patients who did not return this questionnaire were lower educated and had a lower baseline global QoL. The response rate was lowest in intervention refusers (77%) and highest in accepters (93%). Eighty percent of control patients returned the follow-up cohort questionnaire.

#### Physical activity

Mean increase in physical activity level from baseline to 6-month follow-up in the intervention group was 61 min/week (95% CI = 26; 97, Table 2). On average, intervention accepters reported an increase of 67 (95% CI = 26; 109). The increase in refusers was comparable (53 min/week, 95% CI = − 11; 117), but not different from zero. Physical activity in the control group did not change substantially (17, 95% CI = − 16; 51).

**Table 2:** Physical activity level at baseline and changes from baseline to 6 months follow-up

Physical activity level, minutes per week	*n*	Control	*n*	Intervention	*n*	Intervention accepted	*n*	Intervention refused	*n*	Pre- and 12- week post measurements^a^
Baseline, median (IQR)	127	60 (0; 180)	127	60 (0; 120)	67	60 (0; 140)	60	50 (0; 120)	67	75 (0; 210)
Change from baseline to 6 months follow-up, mean (95% CI)	110	17 (− 16; 51)	108	61 (26; 97)	62	67 (26;109)	46	53 (− 11; 117)	61	60 (19; 100)

*CI* confidence interval, *IQR* interquartile range

^a^UMBRELLA Fit pre- and post-intervention measurements in the patients who accepted the intervention

### Quality of life

At baseline, global QoL was comparable to the Dutch general female population (mean = 76.9, SD = 17.9). Within-group changes are shown in Table 3. Compared to control, the exercise intervention did not result in improved global QoL (ITT: 0.8, 95% CI = − 2.2; 3.8, ES = 0.05; IV: 1.3, 95% CI = − 3.9; 6.5) and the summary score (ITT: 0.8, 95% CI = − 1.4; 3.0, ES = 0.06; IV: 1.3, 95% CI = − 2.6; 5.1, Table 4).

**Table 3:** Quality of life, fatigue, and anxiety and depression at baseline and within-group changes

	Baseline		Within-group	
	*n*	Mean (SD)	*n*	Mean change (95% CI)
EORTC-QLQ-C30—Functional scales^a^				
QLQ-C30 summary score; 89.3 (11.9)^b^				
Control	127	87.2 (11.4)	111	6.4 (4.8; 8.0)
Intervention	125	85.5 (11.5)	107	7.1 (5.6; 8.7)
Intervention accepted	66	85.7 (10.2)	61	7.3 (5.1; 9.5)
Intervention refused	59	85.3 (12.9)	46	6.9 (4.7; 9.2)
UMBRELLA Fit measurement^c^	60	85.1 (9.0)	60	4.1 (2.3; 5.9)
Global quality of life; 76.9 (17.9)^b^				
Control	130	79.0 (17.0)	114	0.4 (− 1.9; 2.8)
Intervention	127	76.6 (17.1)	110	1.9 (− 0.4; 4.2)
Intervention accepted	67	78.0 (17.0)	62	1.2 (− 2.0; 4.4)
Intervention refused	60	75.1 (17.2)	48	2.8 (− 0.6; 6.2)
UMBRELLA Fit measurement ^c^	60	76.1 (17.5)	60	8.1 (4.1; 12.0)
Physical functioning; 88.5 (16.0)^b^				
Control	130	87.3 (13.9)	114	0.0 (− 1.7; 1.7)
Intervention	127	86.7 (13.9)	110	− 0.2 (− 2.0; 1.6)
Intervention accepted	67	88.2 (12.4)	62	− 0.1 (− 2.5; 2.3)
Intervention refused	60	85.1 (15.3)	48	− 0.4 (− 3.0; 2.2)
UMBRELLA Fit measurement ^c^	60	86.6 (12.7)	60	3.9 (1.8; 6.0)
Role functioning; 86.3 (22.3)^b^				
Control	130	84.7 (20.2)	114	0.1 (− 3.4; 3.7)
Intervention	127	80.6 (23.3)	110	2.3 (− 1.8; 6.3)
Intervention accepted	67	80.1 (23.8)	62	1.1 (− 4.8; 7.0)
Intervention refused	60	81.1 (22.9)	48	3.8 (− 1.4; 9.1)
UMBRELLA Fit measurement^c^	60	84.2 (18.0)	60	5.3 (0.7; 9.9)
Emotional functioning; 86.6 (17.7)^b^				
Control	130	83.5 (19.7)	114	0.0 (− 3.2; 3.2)
Intervention	127	83.7 (17.7)	110	1.8 (− 1.5; 5.0)
Intervention accepted	67	83.3 (17.1)	62	2.0 (− 2.1; 6.1)
Intervention refused	60	84.1 (18.5)	48	1.5 (− 3.7; 6.8)
UMBRELLA Fit measurement^c^	60	81.3 (14.1)	60	7.1 (3.2; 10.9)
Cognitive functioning; 91.6 (15.8)^b^				
Control	130	81.2 (21.5)	114	0.0 (− 2.8; 2.8)
Intervention	127	83.6 (17.9)	110	1.7 (− 1.3; 4.7)
Intervention accepted	67	83.3 (16.9)	62	1.1 (− 3.2; 5.4)
Intervention refused	60	83.9 (19.2)	48	2.4 (− 1.7; 6.6)
UMBRELLA Fit measurement^c^	60	80.0 (20.5)	60	4.7 (0.8; 8.7)
Social functioning; 92.4 (17.3)^b^				
Control	130	88.6 (17.6)	114	2.2 (− 0.6; 5.0)
Intervention	127	89.1 (19.3)	110	0.5 (− 2.5; 3.5)
Intervention accepted	67	91.5 (15.5)	62	0.5 (− 3.2; 4.3)
Intervention refused	60	86.4 (22.7)	48	0.3 (− 4.6; 5.3)
UMBRELLA Fit measurement^c^	60	88.1 (21.7)	60	6.7 (1.1; 12.3)
MFI-20^d^				
General fatigue				
Control	130	10.6 (4.3)	113	− 0.3 (− 1.0; 0.4)
Intervention	127	11.5 (4.6)	109	− 0.9 (− 1.5; − 0.3)
Intervention accepted	66	11.6 (4.7)	61	− 1.0 (− 1.8; − 0.1)
Intervention refused	61	11.4 (4.7)	48	− 0.8 (− 1.6; 0.0)
UMBRELLA Fit measurement^c^	60	11.1 (3.6)	60	− 2.3 (− 3.3; − 1.3)
Physical fatigue				
Control	130	9.6 (4.1)	113	0.0 (− 0.6; 0.5)
Intervention	127	10.4 (4.8)	108	− 1.2 (− 1.7; − 0.6)
Intervention accepted	66	10.2 (4.9)	61	− 1.2 (− 2.1; − 0.4)
Intervention refused	61	10.7 (4.6)	47	− 1.0 (− 1.8; − 0.3)
UMBRELLA Fit measurement^c^	60	10.5 (3.6)	60	− 2.9 (− 3.7; − 2.0)
Mental fatigue				
Control	130	9.2 (4.3)	113	0.5 (− 0.2; 1.1)
Intervention	127	9.1 (4.0)	108	0.4 (− 0.3; 1.0)
Intervention accepted	66	9.3 (4.1)	61	0.0 (− 0.8; 0.9)
Intervention refused	61	8.9 (3.9)	47	0.8 (− 0.1; 1.7)
UMBRELLA Fit measurement^c^	60	10.1 (3.8)	60	− 1.2 (− 1.9; − 0.5)
Reduced motivation				
Control	130	8.5 (3.9)	114	0.0 (− 0.5; 0.6)
Intervention	127	9.1 (4.2)	109	− 0.2 (− 0.7; 0.3)
Intervention accepted	66	8.7 (4.0)	61	− 0.5 (− 1.0; 0.0)
Intervention refused	61	9.6 (4.3)	48	0.2 (− 0.9; 1.2)
UMBRELLA Fit measurement^c^	60	8.4 (3.2)	60	− 1.1 (− 1.9; − 0.2)
Reduced activity				
Control	130	9.3 (4.3)	113	− 0.3 (− 0.9; 0.3)
Intervention	127	10.3 (4.4)	109	− 0.8 (− 1.3; − 0.2)
Intervention accepted	67	10.1 (4.3)	61	− 1.1 (− 1.8; − 0.3)
Intervention refused	60	10.5 (4.5)	48	− 0.4 (− 1.2; 0.5)
UMBRELLA Fit measurement^c^	60	9.8 (4.0)	60	− 2.2 (− 3.2; − 1.2)
HADS^e^				
Anxiety				
Control	127	5.2 (3.2)	111	0.3 (− 0.2; 0.8)
Intervention	126	4.7 (3.2)	110	0.2 (− 0.4; 0.7)
Intervention accepted	66	4.7 (3.0)	62	0.1 (− 0.5; 0.8)
Intervention refused^c^	60	4.7 (3.5)	48	0.2 (− 0.7; 1.1)
Depression				
Control	127	3.1 (3.4)	111	0.2 (− 0.2; 0.6)
Intervention	126	2.9 (3.3)	110	0.0 (− 0.4; 0.4)
Intervention accepted	66	2.6 (2.8)	62	− 0.8 (− 0.5; 0.4)
Intervention refused	60	3.4 (3.7)	48	0.1 (− 0.7;0.9)

Adjusted for corresponding baseline measures of the outcome, age, time since diagnosis, BMI and education

*CI* confidence interval; *SD* standard deviation

^a^ Scores ranged from 0 to 100 and a higher score indicated better outcomes

^b^ Normative data from the Dutch general female population [38]

^c^ UMBRELLA Fit pre- and 12-week post-intervention measurements in the patients who accepted the intervention

^d^ Scores ranged from 4 to 20 and a higher score indicated more fatigue

^e^ Scores ranged from 0 to 21 and a higher score indicated higher levels of anxious or depressive state

**Table 4:** Effect of the exercise intervention on quality of life, fatigue, and anxiety and depression

	ITT	ES^a^	IV	PSM
	Mean difference (95% CI)		Mean difference (95% CI)	Mean difference (95% CI)
EORTC QLQ-C30—Functional scales^b^				
QLQ-C30 summary score				
Control	Ref			
Intervention	0.8 (− 1.4; 3.0)	0.06	1.3 (− 2.6; 5.1)	0.7 (− 2.6; 3.9)
Global quality of life				
Control	Ref			
Intervention	0.8 (− 2.2; 3.8)	0.05	1.3 (− 3.9; 6.5)	0.2 (− 4.2; 4.6)
Physical functioning				
Control	Ref			
Intervention	− 0.4 (− 2.8; 2.1)	− 0.02	− 0.7 (− 5.0; 3.6)	− 0.3 (− 3.6; 2.9)
Role functioning				
Control	Ref			
Intervention	1.2 (− 3.8; 6.1)	0.05	1.9 (− 6.9; 10.8)	− 0.7 (− 8.0; 6.5)
Emotional functioning				
Control	Ref			
Intervention	2.5 (− 1.3; 6.3)	0.15	4.3 (− 2.4; 11.1)	4.1 (− 1.0; 7.2)
Cognitive functioning				
Control	Ref			
Intervention	3.0 (− 0.9; 6.9)	0.15	5.1 (− 1.8; 12.0)	1.0 (− 4.5; 7.2)
Social functioning				
Control	Ref			
Intervention	− 0.0 (− 4.5; 2.7)	− 0.05	− 1.6 (− 8.1; 4.8)	− 0.5 (− 5.2; 4.3)
MFI-20^c^				
General fatigue				
Control				
Intervention	− 0.4 (− 1.3; 0.5)	− 0.09	− 0.7 (− 2.2; 0.8)	− 0.2 (− 1.4; 1.0)
Physical fatigue				
Control	Ref			
Intervention	− 1.1 (− 1.8; − 0.3)	− 0.24	− 1.8 (− 3.2; 0.5)	− 1.2 (− 2.3; − 0.2)
Mental fatigue				
Control	Ref			
Intervention	− 0.2 (− 1.1; 0.7)	− 0.04	− 0.3 (− 1.8; 1.2)	− 0.4 (− 1.7; 0.9)
Reduced motivation				
Control	Ref			
Intervention	− 0.2 (− 0.9; 0.5)	− 0.05	− 0.4 (− 1.7; 1.0)	− 0.5 (− 1.5; 0.5)
Reduced activity				
Control	Ref			
Intervention	− 0.3 (− 1.1; 0.5)	− 0.07	− 0.6 (− 1.9; 0.8)	− 0.7 (− 1.8; 0.4)
HADS^d^				
Anxiety				
Control	Ref			
Intervention	− 0.1 (− 0.8; 0.6)	− 0.03	− 0.1 (− 1.3; 1.1)	− 0.2 (− 1.2; 0.8)
Depression				
Control	Ref			
Intervention	0.1 (− 1.0; 1.1)	0.03	0.2 (− 0.8; 1.2)	0.0 (− 1.0; 0.9)

Adjusted for corresponding baseline measures of the outcome, age, time since diagnosis, BMI and education

*CI* confidence interval; *ES* standardized effect size; *ITT* intention-to-treat; *IV* instrumental variable; *PSM* propensity score matching

*N*_control_ = 114, *N*_intervention_ = 110, *N*_PSMaccepters/arm_ = 67, *N*_PSMrefusers/arm_ = 62 (some patients do not have an outcome score on a particular scale or domain)

^a^According to Cohen [39], ES ≤ 0.2 = small effect, ES 0.2-0.5 = medium effect, ES ≥ 0.8 = large effect

^b^Scores ranged from 0 to 100 and a higher score indicated better outcomes

^c^Scores ranged from 4 to 20 and a higher score indicated more fatigue

^d^Scores ranged from 0 to 21 and a higher score indicated higher levels of anxious or depressive state

In PS analyses, all covariates were well balanced (i.e., standardized mean differences < 0.1). No differences in the QoL measures were found between actual intervention accepters in the intervention arm and potential accepters in the control arm.

### Fatigue

The intervention group improved in general fatigue (MD = − 0.9, 95% CI = − 1.5; − 0.3), physical fatigue (MD = − 1.2, 95% CI = − 1.7; − 0.6), and “reduced activity” (MD = − 0.8, 95% CI = − 1.3; − 0.2) from baseline to 6 months (Table 3). Intervention accepters showed improvements in these three dimensions, while refusers only improved in physical fatigue (Table 3). In the control group, fatigue did not change over time. When using the pre- and 12-week post-intervention outcomes, intervention patients improved on all fatigue dimensions (Supplemental Table 2).

Compared to controls, patients in the intervention group reported larger, but still small, reductions in physical fatigue (ITT: − 1.1, 95% CI = − 1.8; 0.3, ES = − 0, 24; IV: − 1.8, 95% CI = − 3.2; − 0.5; PS: − 1.2; 95% CI = − 2.3; − 0.2, Table 4). No between-group differences were found for the other fatigue dimensions.

### Anxiety and depression

No differences between the intervention and control group were found on anxiety and depression (ITT: − 0.2, 95% CI = − 1.2, ES = − 0.03; 0.8 and 0.0, 95% CI = − 1.0; 0.9, ES = 0.03, respectively).

### Sensitivity analysis

Fifteen percent of the patients in the intervention group (*n* = 20; whereof 6 intervention accepters and 14 intervention refusers) and twelve percent of the patients in the control group had a missing value on the primary outcome (*n* = 16). Repeating the analyses with missing values on the primary outcome imputed as well as replacing the cohort measurements by the pre- and 12-week post-intervention measurements for patients who did not yet start or not yet complete the intervention before the cohort follow-up measurement, yielded comparable results (Supplemental Material). However, the intervention group reported larger, but still small reductions in general fatigue and reduced activity compared to the control group when using the 12-week post-intervention outcomes (− 0.9, 95% CI = − 1.7; − 0.0 and − 0.9, 95% CI = − 1.7; − 0.1 respectively).

No differences in QoL and fatigue were found between intervention refusers and potential refusers (control group; data not shown).

## Discussion

This study showed no effect on QoL, and a small but beneficial effect of a 12-week exercise intervention on physical fatigue in patients after breast cancer treatment. More than half of the patients performing no or little physical activity started exercising. The TwiCs design provided insights in the effect of refusing an intervention offer. Interestingly, in refusers, we observed slightly higher levels of physical activity, QoL and fatigue compared to the control group, which was not statistically significant when formally tested. This may imply that offering an intervention and actively refusing the offer might induce lifestyle changes.

In contrast to a systematic review reporting positive effects of exercise on QoL in breast cancer patients, we found no effect [8]. In UMBRELLA Fit, eligibility of patients was based on a physically inactive lifestyle and not on a low QoL. QoL of the study population was comparable to the Dutch general female population [38]. Therefore, room for improvement in QoL was limited. In a previous trial from our group in which patients experienced at least three problems on QoL domains, exercise indeed led to a relevant increase in QoL compared to control [40].

In line with a meta-analysis that showed that physical fatigue is the most sensitive fatigue dimension in exercise-oncology trials, we found a positive effect of exercise on physical fatigue [41]. In UMBRELLA cohort patients, QoL decreased during treatment and recovered in the year after treatment, whereas fatigue remained high as compared to a female Dutch reference population, even 18 months after treatment (unpublished observation), and is thus a symptom that indeed needs attention.

### Implications of the TwiCs design for effect estimation

Because patients may refuse the intervention, it is recommended from simulation studies to perform both ITT and IV analyses to take non-acceptance into account when estimating the ‘real’ intervention effect [29, 30]. The ITT analysis showed the effect of offering an exercise intervention, which resembles clinical practice, but it may dilute the treatment effect dependent on the extent of non-acceptance. The IV analysis took the chance of non-acceptance into account. To check its robustness, we also performed PS analyses and compared intervention accepters with potential accepters in the control group. All (sensitivity) analyses yielded comparable results, indicating that the impact of intervention refusal on the effect size seems small. Although no differences in outcomes were found between patients who refused the intervention and potential refusers in the control group, we cannot exclude that there was an effect of offering the intervention on outcomes, which then also affects the IV and PS analyses. However, analyses were not powered to detect an effect in refusers; therefore, these results are explorative.

Due to the design, we experienced that it was sometimes challenging to schedule the intervention in between the two cohort measurements. Consequently, 35 intervention accepters (of 68, 51%) had not yet completed the intervention or started (*n* = 3/68, 4%) with the intervention when the endpoint was evaluated. Therefore, the effects may be underestimated. As a sensitivity analysis, we replaced the cohort measurements by the pre- and 12-week post-intervention measurements for these patients and this yielded slightly larger effects. We also considered using the first cohort measurement after the study end for patients who did not yet complete the intervention before the planned cohort outcome measurement. These could then be matched to random control patients. However, this could introduce a risk of immortal time bias since both groups need to survive until the extended measurement point. Intervention and control patients could be matched on baseline variables, yet this would require selection based on post-intervention variables (notably survival until end of follow-up), thus potentially introducing a selection bias. Given these considerations, we did not conduct these sensitivity analyses. We recommend being stringent in intervention planning and taking a suitable follow-up period when designing a TwiCs.

We observed that the 6-month changes were smaller than the 12-week post-intervention changes. First, as described above, not every patient completed the intervention before the endpoint was evaluated. Second, cohort measurements were not obtained in a trial setting, whereas patients may have completed the 12-week post-intervention questionnaire with their trial participation in mind. Third, the 6-month cohort outcomes might present a ‘long-term’ effect of the intervention. Therefore, long-term cohort outcomes might be representative for the (long-term) real world effects.

### Implications for clinical practice

Because the TwiCs consent procedure better reflects clinical practice than in conventional trials, this study provided important insight into intervention uptake and reasons for refusal. Because we used the TwiCs design, we learned that more than half of the patients performing no or little physical activity started exercising and subsequent adherence was high. Therefore, health care providers should not hesitate to motivate patients to become physically active. Yet, almost half of the patients refused the offer of the exercise intervention, even when it was free of costs. Based on their reasons for refusal, one can consider adaptations to, or alternatives for the intervention, to reinforce uptake in intervention refusers, e.g., offering these patients a home-based exercise program.

## Conclusion

In this TwiCs, more than half of the inactive patients with breast cancer accepted the offer of a 12-week supervised exercise intervention. We found no effects of offering an exercise program on QoL, which was already high at baseline. For physical fatigue, we found small but beneficial exercise effects. Applying the TwiCs design appeared feasible. The use of the TwiCs design could dilute effect sizes because of intervention refusal. In this TwiCs, the impact of intervention refusal seemed small because of consistent results of the ITT and IV analysis (taking non-compliance into account). In addition, the use of cohort measurements for effect estimation may have diluted the effect size. Therefore, we recommend careful intervention planning in-between the cohort measurements that will be used as pre- and post-intervention measurement.

## Data Availability

The datasets generated and/or analyzed during the current study are available from the corresponding author on reasonable request.

## Code availability

The R code is available from the corresponding author upon reasonable request.
